# Supplementary material for: OncoTherad® (MRB-CFI-1) Nanoimmunotherapy: A Promising Strategy to Treat Bacillus Calmette–Guérin-Unresponsive Non-Muscle-Invasive Bladder Cancer: Crosstalk among T-Cell CX3CR1, Immune Checkpoints, and the Toll-Like Receptor 4 Signaling Pathway
Source: Int J Mol Sci. 2023 Dec 15;24(24):17535. doi: 10.3390/ijms242417535 (PMC10743608; doi:10.3390/ijms242417535)
Supplement: Supplementary file 1 [file ijms-24-17535-s001.zip › ijms-2736106-supplementary.pdf]

SUPPLEMENTARY DATA

OncoTherad® (MRB-CFI-1) Nanoimmunotherapy: A Promising Strategy to Treat Bacillus Calmette–Guérin-Unresponsive Non-Muscle-Invasive Bladder Cancer: Crosstalk among T-Cell CX3CR1, Immune Checkpoints, and the Toll-Like Receptor 4 Signaling Pathway

João Carlos Cardoso Alonso [1,2,*], Bianca Ribeiro de Souza [3], Ianny Brum Reis [4], Gabriela Cardoso de Arruda Camargo [1], Gabriela de Oliveira [1], Maria Izabel de Barros Frazão Salmazo [1], Juliana Mattoso Gonçalves [1], José Ronaldo de Castro Roston [1], Paulo Henrique Ferreira Caria [1], André da Silva Santos [1], Leandro Luiz Lopes de Freitas [5], Athanase Billis [5], Nelson Durán [1] and Wagner José Fávaro [1,*]

[1] Laboratory of Urogenital Carcinogenesis and Immunotherapy (LCURGIN), Universidade Estadual de Campinas (UNICAMP), Campinas 13083-865, São Paulo, Brazil; gcacamargo0@gmail.com (G.C.d.A.C.); gabriela.ufscar@gmail.com (G.d.O.); mabelfrazao@hotmail.com (M.I.d.B.F.S.); jumattosogg@gmail.com (J.M.G.); rroston@uol.com.br (J.R.d.C.R.); phcaria@unicamp.br (P.H.F.C.); andre.s.s@me.com (A.d.S.S.); nelsonduran1942@gmail.com (N.D.)

[2] Paulínia Municipal Hospital, Paulínia 13140-000, São Paulo, Brazil

[3] Obstetrics & Gynecology Department, Ovarian Cancer Research Group University of British Columbia, Vancouver, BC V6Z 2K8, Canada; bianca.ribeiro@ubc.ca

[4] Diagnosis and Surgery Department, Dentistry School, São Paulo State University (UNESP), Araraquara 14801-903, São Paulo, Brazil; iannybrumreis@yahoo.com.br

[5] Pathology Department, Medical School, Universidade Estadual de Campinas (UNICAMP), Campinas 13083-888, São Paulo, Brazil; leandrollfreitas@gmail.com (L.L.L.d.F.); athanase@unicamp.br (A.B.)

* Correspondence: jocaalonso1@gmail.com (J.C.C.A.); favarowj@unicamp.br (W.J.F.)

Table S1: Tissue therapeutic responses (non-neoplastic) to OncoTherad® (MRB-CFI-1) nanoimmunotherapy at the end of the 24-month follow-up.

Therapeutic Responses	N	%
**Normal Histology**	03/44	6.8
**Non-Neoplastic Histological Changes**		
Follicular cystitis	35/44	79.6
Chronic cystitis	03/44	6.8
Erosive cystitis + granulation tissue	02/44	4.5
Flat urothelial hyperplasia + reactive atypia	01/44	2.3

Table S2: Comparison of biochemical serological parameters before and after intravesical and intramuscular OncoTherad® (MRB-CFI-1) treatment.

Parameters		Treatment		
		**Before OncoTherad®**	**After OncoTherad®**	***P*-Value**
**Glucose (mg/dL)**	**N**	44	44	
	**Average**	92.0	96.5	
	**SD**	5.3	3.2	
	**Maximum**	103.0	118.0	> 0.05
	**Median**	91.0	94.0	
	**Minimum**	82.0	84.0	
**Hemoglobin (g/dL)**	**N**	44	44	
	**Average**	14.3	14.3	
	**SD**	2.4	2.2	
	**Maximum**	18.0	17.9	> 0.05
	**Median**	14.4	14.6	
	**Minimum**	10.5	10.6	
**Leukocytes (mm^3)**	**N**	44	44	
	**Average**	8,199.2	7,505.5	
	**SD**	2,542.4	2,709.0	
	**Maximum**	13,400.0	12,850.0	> 0.05
	**Median**	8,766.0	7,855.0	
	**Minimum**	3,601.0	3,084.0	
**Platelets (mm^3)**	**N**	44	44	
	**Average**	230,543.5	239,860.2	
	**SD**	97,800.0	87,400.2	
	**Maximum**	420,800.0	421,900.0	> 0.05
	**Median**	208,900.0	237,550.0	
	**Minimum**	118,630.0	116,815.0	
**Aspartate transaminase (AST) (U/L)**	**N**	44	44	
	**Average**	26.8	29.4	
	**SD**	7.0	12.3	
	**Maximum**	42.1	72.4	> 0.05
	**Median**	28.4	27.9	
	**Minimum**	15.8	15.9	
**Alanine transaminase (ALT) (U/L)**	**N**	44	44	
	**Average**	20.6	22.7	
	**SD**	9.7	11.0	
	**Maximum**	46.4	49.8	> 0.05
	**Median**	19.8	21.8	
	**Minimum**	7.0	7.5	
**Urea (mg/dL)**	**N**	44	44	
	**Average**	38.2	40.1	
	**SD**	5.6	10.0	
	**Maximum**	51.0	72.5	> 0.05
	**Median**	38.9	38.0	
	**Minimum**	25.0	27.0	
**Creatinine (mg/dL)**	**N**	44	44	
	**Average**	0.9	1.0	
	**SD**	0.2	0.4	
	**Maximum**	1.4	2.1	> 0.05
	**Median**	0.8	0.9	
	**Minimum**	0.6	0.6	
**Gamma-glutamyl transferase (GGT) (U/L)**	**N**	44	44	
	**Average**	29.9	39.0	
	**SD**	11.7	17.3	
	**Maximum**	57.0	73.0	> 0.05
	**Median**	27.5	35.5	
	**Minimum**	11.0	10.5	

SD= Standard deviation. Reference values: Glucose: 65 – 99 mg/dL; Hemoglobin: 12 – 18 g/dL; Leukocytes: 4,000 – 11,000/mm^3; Platelets: 150,000 – 400,000/mm^3; AST: until 38 U/L; ALT: until 37 U/L; Urea: 15 – 50
mg/dL; Creatinine: 0.5 – 1.2 mg/dL; GGT: 8 – 61 U/L.

Table S3: Primary antibodies for Immunohistochemistry (IHC) and Western Blotting (WB).

Antibody	Code	Distributor	Dilution
Rabbit polyclonal anti-TLR4	ABBI-251111	Abbiotec, EUA	IHC: 1:200
Mouse monoclonal anti-TRIF	sc-514384	Santa Cruz Biotechnology, EUA	IHC: 1:150
Mouse monoclonal anti-TBK1	sc-398366	Santa Cruz Biotechnology, EUA	IHC: 1:200
Mouse monoclonal anti-IRF3	sc-33641	Santa Cruz Biotechnology, EUA	IHC: 1:200
Mouse monoclonal anti-IFN-γ	sc-12755	Santa Cruz Biotechnology, EUA	IHC: 1:100
Mouse monoclonal anti-CX3CR1	sc-377227	Santa Cruz Biotechnology, EUA	IHC: 1:150; WB: 1:400
Mouse monoclonal anti-FOXP3	sc-53876	Santa Cruz Biotechnology, EUA	IHC: 1:300
Mouse monoclonal anti-PD-L1	sc-518027	Santa Cruz Biotechnology, EUA	IHC: 1:150
Mouse monoclonal anti-CTLA-4	sc-376016	Santa Cruz Biotechnology, EUA	IHC: 1:200
Rabbit polyclonal anti-iNOS	ab3523	Abcam, EUA	IHC: 1:150
Mouse monoclonal anti-�-Actina	A5441	MilliporeSigma, EUA	WB: 1:2000

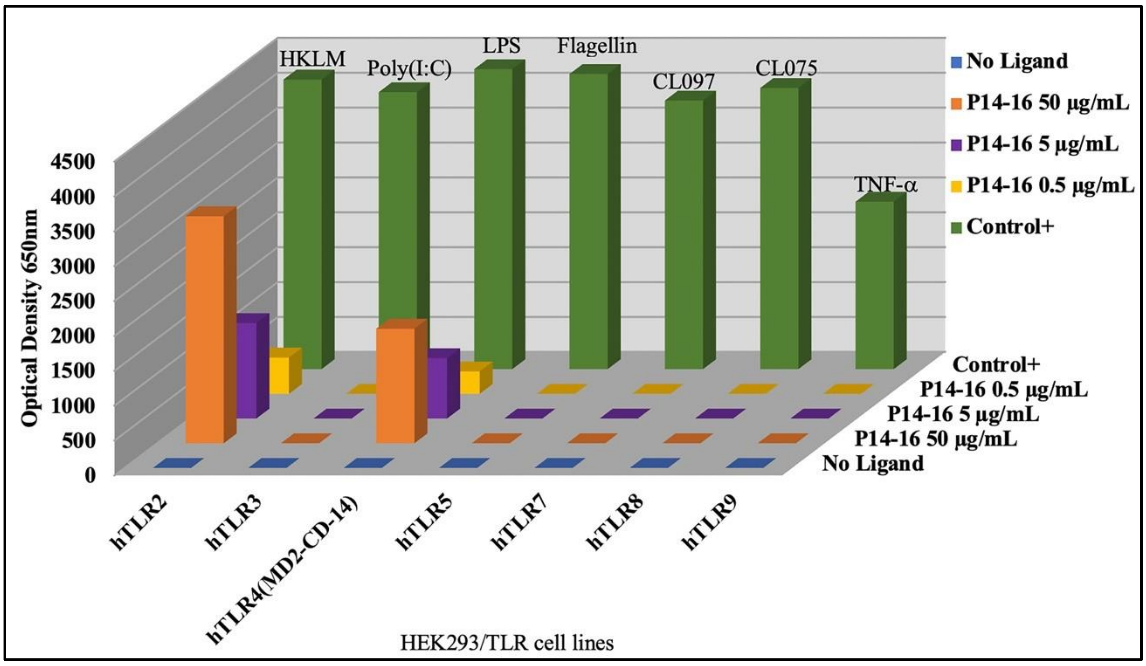

Figure S1: Human TLR ligand screening – P14-16 protein.

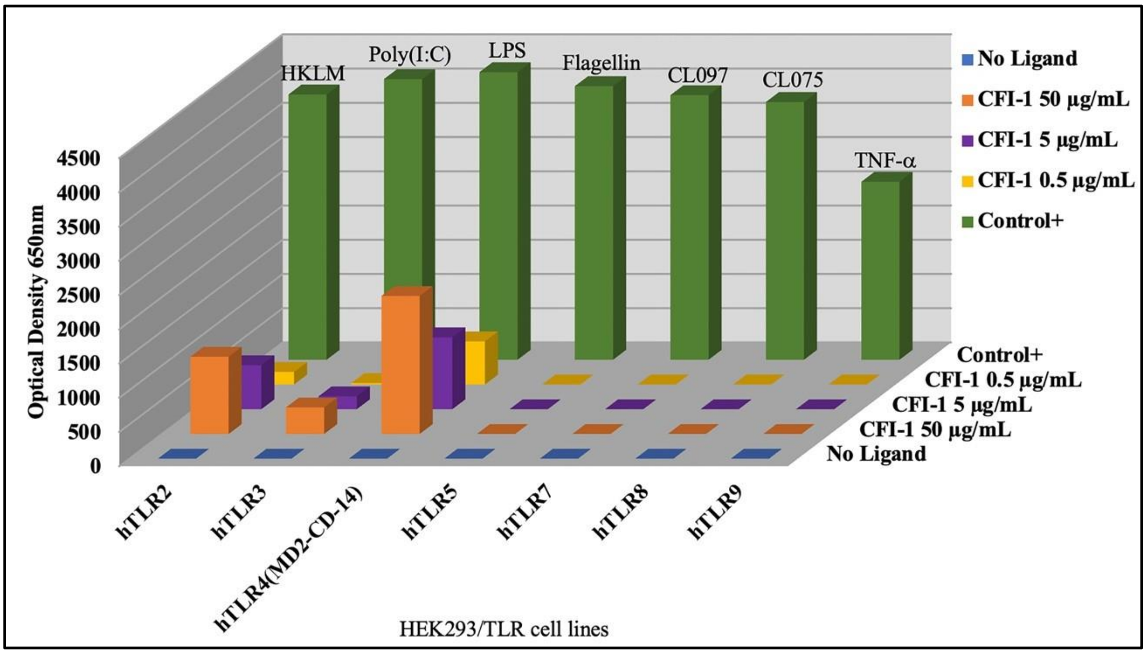

Figure S2: Human TLR ligand screening – Inorganic nanostructured complex-1 (CFI-1).

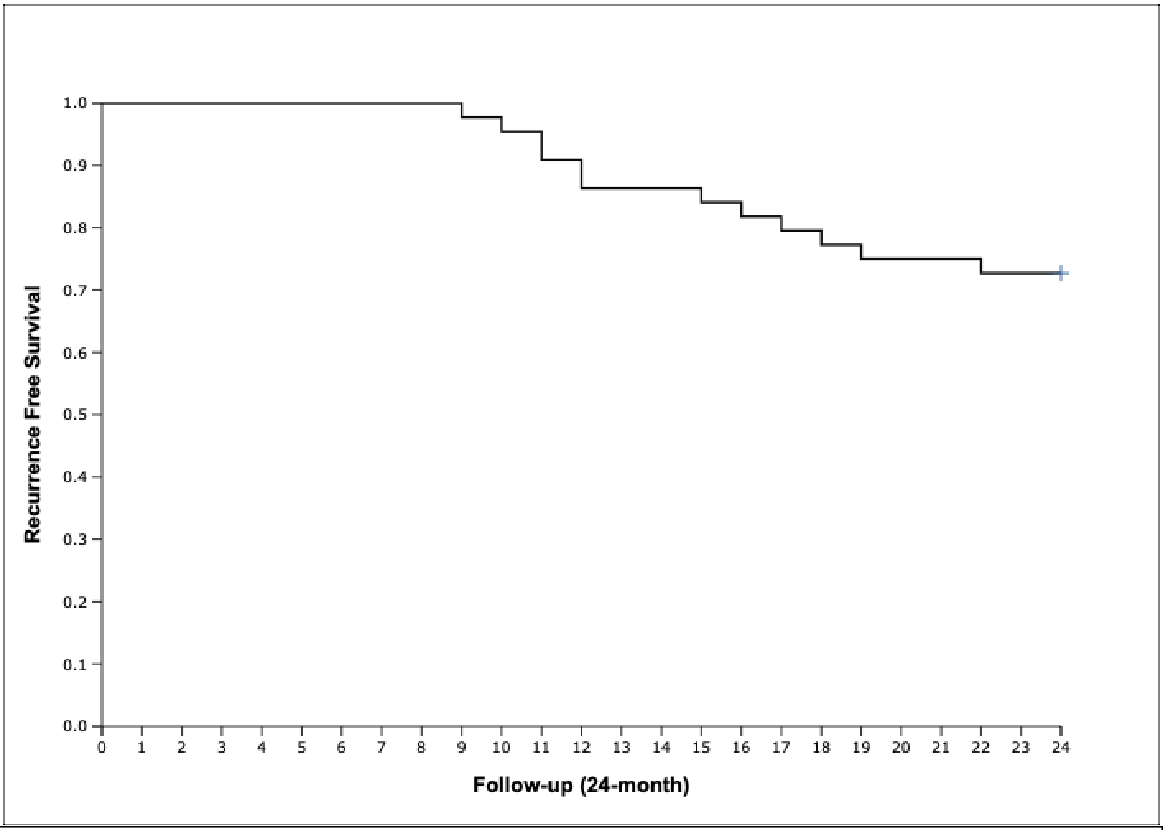

Statistical summary for Recurrence-Free Survival

Estimate of percentiles

Percentil	Estimate Point	95% confidence interval [Inferior	Superior)
75	.	.	.
50	.	.	.
25	.	09.0000	.

Average	Standard error
21.4	0.72

Total	Failed	Censored	Percent Censored
44	12	32	72.7

Figure S3: The Kaplan-Meier curve for Recurrence Free Survival in the 24-month follow-up of OncoTherad® (MRB-CFI-1) treatment.

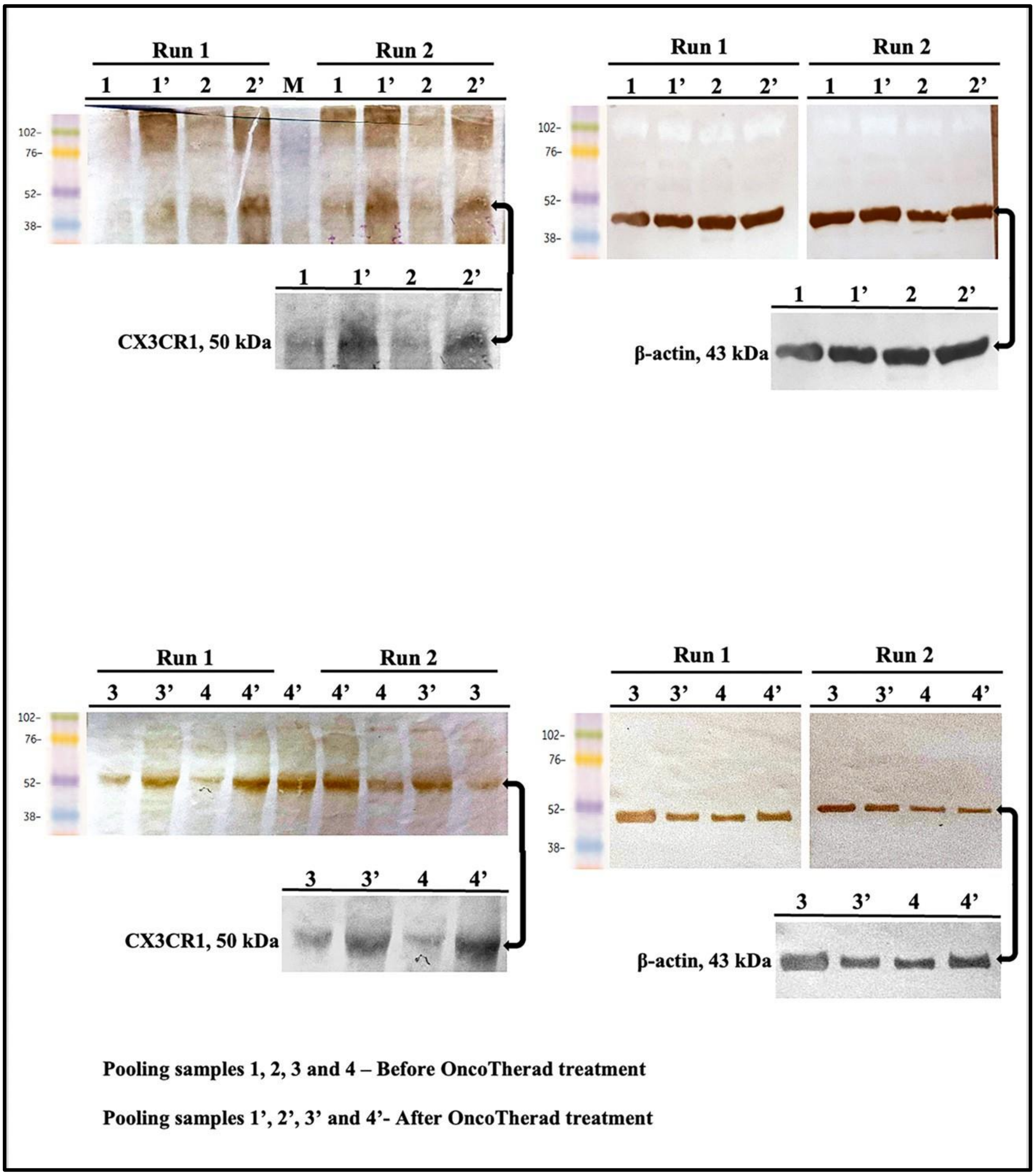

Figure S4: Original immunoblots of CX3CR1 and �-actin (endogenous positive control). The immunoreactivity of the bands was visualized by incubating them with DAB (diaminobenzidine) chromogen. The samples from the 20 patients, both before and after OncoTherad® (MRB-CFI-1) treatment, were grouped into four pools (each containing n=5 samples). Immunoblots were conducted in duplicate (run 1 and run 2). M= Protein Molecular Weight.
